# Polymorphisms of Tumor Necrosis Factor Alpha in Moroccan Patients with Gastric Pathology: New Single-Nucleotide Polymorphisms in TNF-*α* ^−193^ (G/A)

**DOI:** 10.1155/2015/143941

**Published:** 2015-10-04

**Authors:** A. Essadik, H. Jouhadi, T. Rhouda, S. Nadifiyine, A. Kettani, F. Maachi

**Affiliations:** ^1^Laboratory of Pathology-Digestif-Oncology, Pasteur Institute of Morocco, 20360 Casablanca, Morocco; ^2^Laboratory of Biology and Health, Molecular Modeling and Quality Control Team, Faculty of Sciences Ben M'sik, University Hassan II, 7955 Casablanca, Morocco; ^3^Department of Radiotherapy Oncology, Ibn Rochd University Hospital, 20000 Casablanca, Morocco; ^4^Laboratory of Molecular Genetics and Pathophysiology, Faculty of Sciences Ben M'sik, University Hassan II, 7955 Casablanca, Morocco

## Abstract

Polymorphisms in tumor necrosis factor alpha (TNF-*α*) gene are emerging as key determinants of gastric diseases. The TNF-*α* ^−308^ (G/A) and TNF-*α* ^−238^ (G/A) single-nucleotide polymorphisms SNPs are the most extensively studied. However, all these studies are conducted in Caucasian and Asian populations. Thus, for the first time in Africa, we sought to investigate whether polymorphisms in TNF-*α* gene were associated with the development of gastric pathology in Morocco. Two SNPs located in the promoter region (positions −308 and −238) in TNF-*α* gene were genotyped in 244 individuals (170 patients and 74 healthy controls). Odds ratios (ORs) and 95% confidence intervals (CI) were estimated using logistic regression analysis. The TNF-*α* ^−238^ (G/A) genotype was significantly associated with a high risk of gastritis and gastric cancer (GC) (*P* = 0.001 and *P* = 0.002, resp.). Furthermore, a new polymorphism located in the promoter region at position −193 in TNF-*α* gene was identified. The distribution of this SNP was markedly different in patients suffering from ulcers. The association between TNF-*α* ^−193^ (G/A) genotype and high risk of ulcer was significant (*P* = 0.03). These results suggest that the TNF-*α* ^−193^ (G/A) allele has a protective function against gastric cancer by developing ulcer.

## 1. Introduction

Gastric cancer (GC) is the fourth most common malignancy worldwide [1], with a frequency that varies greatly across different geographic locations [2]. Patients infected with* Helicobacter pylori *(*H. pylori*) are at increased risk for developing GC [3]. It is now known that* H. pylori* plays a major role in gastric carcinogenesis [4] through physiological and histological changes that it induces in the stomach mucosa [5, 6]. However, a striking difference exists between the number of infected individuals and the number that goes on to develop GC [6]. Hence progression toward gastric cancer is likely depending on a multifactorial etiology, combining effects of bacterial pathogenicity, host susceptibility, and environmental factors [3, 6, 7].

Host genetic factors are emerging as key determinant of diseases for many cancers [8]. Genetic variations in proinflammatory and anti-inflammatory cytokines genes influence individual response to carcinogenic exposures. Polymorphisms in proinflammatory cytokine genes, especially tumor necrosis factor (TNF-*α*) and its receptor, are associated with an increased risk of GC [9]. Indeed, SNPs in regulatory regions of TNF-*α* play a crucial role on gene transcription levels [10]. Several SNPs have been identified in the TNF-*α* gene, mainly in the 5′-promoter region [11]. Two SNPs in positions −308 and −238 have been most frequently evaluated for association with gastric cancer. The TNF-*α* ^−308^ (G/A) is associated with an increased production of TNF-*α* [3], which is a central mediator of the immune response and shares many biological properties with IL-1. The function and significance of TNF-*α* ^−238^ (G/A) are less clear, but, because a putative repressor site is located in a 25-base stretch that includes position −238, this polymorphism may be functional [12, 13]. The TNF-*α* ^−308^ (G/A) and TNF-*α* ^−238^ (G/A) single-nucleotide polymorphisms SNPs are the most extensively studied. However, all these studies are conducted in Caucasian and Asian populations. In Africa, up to today, no particular study was interested in the association between polymorphism in the promoter of the TNF-*α* gene and high risk of GC. Thus, the aim of the present work was to study the frequency of SNPs in TNF-*α* gene in Moroccan patients with gastritis, ulcer, and gastric cancer.

## 2. Patients and Methods

### 2.1. Patients and Controls

In this case control study, a total of 244 GC patients were enrolled (93 patients with GC, 56 patients with chronic gastritis, and 21 patients suffering from ulcer and controls group consisted of 74 healthy volunteers, without any gastric disorders). A comprehensive set of sociodemographic, clinical, and laboratory data have been collected. Written informed consent was obtained for all subjects.

### 2.2. Genotyping Methods

To allow simultaneous determination of genotypes at the TNF-*α* ^−308^ (G/A) and TNF-*α* ^−238^ (G/A) SNPs, a 266-bp fragment of the 5′ flanking region of the TNF alpha gene was amplified from genomic DNA extracted from blood samples, by polymerase chain reaction (PCR). A total of 0.2 *μ*g of genomic DNA was used, along with 200 *μ*M of dNTP, 1.5 m M of MgCl_2_, 0.5 *μ*M of primers (Table 1), and 1 unit of Taq polymerase in standard PCR buffer to a final volume of 20 *μ*L. PCR conditions were as follows: initial denaturation 94°C for 10 minutes, followed by 35 cycles of 94°C for 30 seconds, 57°C for 30 seconds, 72°C for 30 seconds, and then a final extension for 7 min at 72°C. The reaction volume and conditions for the PCR were essentially the same for TNF-*α* ^−238^ (G/A) PCR, except, for the annealing temperature, it was 55°C.

The PCR products were analyzed by electrophoresis on 2% agarose gel and purified using 0.5 *μ*L of exonuclease, 1 *μ*L alkaline phosphatase, 0.5 *μ*L of buffer in a final volume of 7 *μ*L, and 10–50 ng of PCR product and incubated at 37°C for 30 min and 80°C for 15 min. DNA cycle sequencing was carried out in 20 *μ*L reactions using 5 *μ*L of purified PCR product, 1 *μ*L of 3.2 pmol primer, and 1 *μ*L and ABI Prism BigDye 3.1. The forward and reverse sequencing primers were the same as those used in the respective PCR amplifications, in separate sequencing reactions. Conditions for the sequencing of PCR products were 25 cycles with 10 seconds at 96°C, 5 seconds at 50°C, and 4 min at 60°C. Residual dideoxy terminators were removed by ethanol precipitation and sequences were analyzed on an Applied Biosystems 377 DNA sequencer.

### 2.3. Statistical Analysis

Evidence for deviation from Hardy-Weinberg equilibrium of alleles at individual loci was assessed by exact tests. Differences in the proportion of the TNF-*α* ^−308^ (G/A) and TNF-*α* ^−238^ (G/A) genotype frequencies between cases and controls were assessed by test khi^2^ statistics. Crude odds ratios (ORs), adjusted ORs, and the simultaneous effect of the genetic polymorphisms with 95% confidence intervals (CIs) were estimated by logistic regression analysis. ORs and unconditional logistic regression models were computed using the program Epi Info 2002 (available: ftp://ftp.cdc.gov/pub/Software/epi_info/). Differences were considered to be significant at a *P* value < 0.05. All statistical tests were 2-sided.

## 3. Results

Our study is conducted in a based population cohort with 244 participants. The healthy controls served to compare the frequency of mutations between the patient groups and the general population. In our study, we were initially interested by TNF-*α* polymorphisms at positions −308 and −238. Despite the disparity of −308 SNPs in the world, it is absent in our population. However, the analysis of the TNF-*α* promoter from position −106 to position −372 revealed the presence of a new mutation in position −193. This is a transition from guanine to adenine G/A. The frequency of occurrence of the TNF-*α* ^−193^ A allele in our population is 17.35% in patients suffering from gastric diseases and 13.51% in controls. The distribution of the three genotypes GG, GA, and AA is irregular between controls and patients. The frequencies of the TNF-*α* ^−193^ A allele and the GA + AA genotypes were slightly higher in patients suffering from ulcers. In addition, ulcer presence was significantly more frequent in A carrier allele group TNF-*α* ^−193^ (G/A) (Table 2).

In addition, we demonstrated a striking association between SNP −238 in TNF-*α* gene with gastritis and adenocarcinoma. Taken together, these results strongly support our hypothesis suggesting that TNF-*α* ^−238^ (G/A) polymorphism may be involved in gastritis susceptibility, which progresses in adenocarcinoma (Table 3).

## 4. Discussion

TNF-*α* is an important immune mediator in the inflammatory response and has been suggested to be an endogenous tumor promoter in human carcinogenesis [14, 15]. Some specific polymorphisms might interfere with transcription of the TNF-*α* gene, influencing the circulating level of TNF-*α* and thus increasing susceptibility to gastric pathology outcome to* H. pylori* infection [3, 16, 17]. The aim of our study was to associate TNF-*α* polymorphism to the high risk of gastric diseases.

Currently, several molecular epidemiological studies have been conducted to investigate the association between the SNPs in TNF-*α* and gastric cancer risk [18]. Some common SNPs were identified in the TNF-*α* gene, specially TNF-*α* ^−308^ (G/A) and TNF-*α* ^−238^ G/A, which were extensively studied [19, 20]. However, all these studies were performed on Caucasians or Asian populations. To our knowledge, no study has focused on the relationship between TNF-*α* polymorphisms in gastric cancer in African populations. In case of our study, we identified a new SNP located in the position −193 never shown before, using sequencing. This is a transition from guanine to adenine (G/A). The TNF-*α* ^−193^ (G/A) has been significantly associated with high risk of ulcer. This result suggests that the TNF-*α* ^−193^ (G/A) allele has a protective function against gastric cancer by developing ulcer. Indeed, Correa has established a cascade of events leading to GC more than 20 years ago. Chronic inflammation induced by Hp may progress further through the premalignant stages of gastric atrophy, intestinal metaplasia, dysplasia, and finally adenocarcinoma [21]. The relation between peptic ulcer and gastric carcinoma has long been a matter of controversy. By contrast, duodenal ulcer disease has often been inversely associated with gastric cancer. Some studies were conducted to investigate the role of ulcer in gastric adenocarcinoma development. They concluded that gastric ulcer was a protective factor against GC. Besides, the majority of studies done on the polymorphism of TNF-*α* are based on the research of known SNPs using PCR-RFLP or sometimes the Taq Man technics. Therefore, we limited ourselves to look for new SNPs that may be specific to certain populations. Proof of this study's originality identifies a novel SNP TNF-*α* ^−193^ (G/A) by using sequencing technics.

To date, the association between TNF-*α* ^−308^ (G/A), TNF-*α* ^−238^ (G/A) polymorphism, and gastric cancer was a paradox [12]. There are a large number of studies focused on the association between TNF-*α* ^−308^ G/A polymorphism and gastric cancer. The results of these studies remain inconclusive or inconsistent. Example of TNF-*α* ^−308^ (G/A), while our study has not found association between this SNP and gastric pathology like Japan [22], China [23], and Korea [24], other studies confirm that relationship [25, 26]. Moreover, some meta-analysis summarizing all these results studies, identified the TNF-*α* polymorphisms as a risk factor for gastric cancer in Caucasian populations, but not in Asian populations [9, 27, 28]. The most recent meta-analysis published in 2014 has illuminated this question [12]. They demonstrated that the TNF-*α* ^−308^ (G/A) polymorphism was correlated with gastric cancer risk in Caucasians, but not in East Asians and other ethnic groups. Likewise, Gorouhi et al. showed that the statistically significant association between TNF-*α* ^−308^ (G/A) and gastric cancer was limited to western populations [9]. This association showed little heterogeneity and remained consistently strong when analyses were limited to anatomic and histological subtypes of gastric cancer, or limited to studies in which genotype frequencies were in Hardy-Weinberg equilibrium or limited to larger studies.

Nonetheless, TNF-*α* ^−238^ (G/A) is the second commonly studied SNP site following −308 among all polymorphisms located in the promoter region of TNF-*α* gene [19, 29]. Its function and significance are still less clear, but, because a putative repressor site is located in a 25-base stretch that includes position −238, this polymorphism may be functional [9]. In our study, the TNF-*α* ^−238^ was significantly associated with high risk of gastritis and GC. These results are similar to those found by others [30, 31]. Yu et al. have published a meta-analysis in 2013 [19]. That study showed a significant association between TNF-*α* ^−238^ and high risk of GC. In contrary, another meta-analysis showed no significant association concerning TNF-*α* ^−238^ frequency for genotypes G/G, A/A, and G/A [6, 15, 20]. However, no integrated analysis has been made to get a definitive conclusion of whether TNF-*α* ^−238^ (G/A) is associated with GC [19]. To our knowledge, among all publications, studies investigated the association between TNF-*α* ^−238^ polymorphism and risk of gastric diseases yielded contradictory results.

## Figures and Tables

**Table 1:** Technical data for the analysis of TNF-*α* polymorphisms.

Gene	Location	Primer pairs used	Size of fragments
TNF-*α*	Promoter	F: 5′-(−372) GAAGGAAACAGACCACAGAC3′-(−353)	266 bp
		R: 5′-(−106) ATCTGGAGGAAGCGGTAGTG3′-(−128)	

**Table 2:** Association of a new *single-nucleotide polymorphisms* TNF-*α* ^−193^ (G/A) with gastric pathology.

	Genotypes	Genotyping frequency	MAF	OR	IC	*P* value

Healthy patients	G/G	56 (75.68)	0.02	—	—	—
	A/G + A/A	16 (21.62)				

Adenocarcinoma	G/G	77 (82.8)	0.12	0.64	(0.30–1.38)	0.25^NS^
	A/G + A/A	16 (17.2)				

Gastritis	G/G	44 (78.6)	0.18	0.84	(0.36–1.94)	0.69^NS^
	A/G + A/A	12 (21.4)				

Ulcer	G/G	11 (52.38)	0.40	2.82	1.03–7.74	0.03^S^
	A/G + A/A	10 (47.62)				

Data are expressed as %. TNF-*α*: tumor necrosis factor alpha; A/G: adenine/guanidine; MAF: minor allele frequency; S: significant; NS: not significant ORs with 95% CI; and *P* values were calculated for wild/wild genotype versus wild/mutant and mutant/mutant genotypes.

**Table 3:** Genotype frequencies of TNF-*α* ^−238^ (G/A) among cases and controls and the association with gastric pathology.

	Genotypes	Genotyping frequency	MAF	HWE	*P* value

Controls	G/G	58 (78.4)	0.11	1.05^E^
	G/A	15 (20.3)		
	A/A	1 (1.3)		

Gastritis	G/G	55 (98.2)	0.01	0.016^E^	0.001^S^
	A/G	1 (1.8)			
	A/A	0			

Ulcer	G/G	20 (95.2)	0.02	0.19^E^	0.07^NS^
	A/G	1 (4.8)			
	A/A	0			

Adenocarcinoma	G/G	88 (94.6)	0.03	0.12^E^	0.002^S^
	A/G	5 (5.4)			
	A/A	0			

Data are expressed as %. TNF-*α*: tumor necrosis factor alpha; A/G: adenine/guanidine; MAF: minor allele frequency; HWE: Hardy-Weinberg equilibrium; E: population in equilibrium for TNF-*α* ^−238^ polymorphism; S: significant; NS: not significant ORs with 95% CI; and *P* values were calculated for wild/wild genotype versus wild/mutant and mutant/mutant genotypes.
